# Low Spatial Frequency Bias in Schizophrenia is Not Face Specific: When the Integration of Coarse and Fine Information Fails

**DOI:** 10.3389/fpsyg.2013.00248

**Published:** 2013-05-06

**Authors:** Vincent Laprevote, Aude Oliva, Anne-Sophie Ternois, Raymund Schwan, Pierre Thomas, Muriel Boucart

**Affiliations:** ^1^Centre d’Investigation Clinique-INSERM 9501, CHU NancyNancy, France; ^2^Centre de Soins, d’Accompagnement et de Prévention en Addictologie, CHU NancyNancy, France; ^3^Computer Science and Artificial Intelligence Laboratory, Massachusetts Institute of TechnologyCambridge, MA, USA; ^4^Pôle de Psychiatrie, Centre Hospitalier Régional Universitaire de LilleLille, France; ^5^Faculté de Médecine, Université LorraineNancy, France; ^6^Laboratoire de Neuroscience Fonctionnelle et Pathologies EA 4559, Université Lille-Nord de FranceLille, France

**Keywords:** schizophrenia, spatial frequency, vision, hybrid image, magnocellular, object

## Abstract

Studies have shown that patients with schizophrenia exhibit visual processing impairments, particularly regarding the processing of spatial frequencies. In a previous work, we found that, compared to healthy volunteers, patients were biased toward low spatial frequencies (LSF) to identify facial expression at a glance. Given the ubiquity of faces in visual perception, it remains an open question whether the LSF bias is face specific or also occurs with other visual objects. Here, 15 patients with schizophrenia and 11 healthy control adults performed a categorization task with hybrid stimuli. These stimuli were single images consisting of two different objects, a fruit and an animal, each in a specific spatial frequency range, either low (LSF) or high (HSF). Observers were asked to report if they saw an animal or a fruit. The reported category demonstrated which spatial scale was preferentially perceived in each trial. In a control experiment, participants performed the same task but with images of only a single object, either a LSF or HSF filtered animal or fruit, to verify that participants could perceive both HSF or LSF when presented in isolation. The results on the categorization task showed that patients chose more frequently LSF with hybrid stimuli compared to healthy controls. However, both populations performed equally well with HSF and LSF filtered pictures in the control experiment, demonstrating that the LSF preference found with hybrid stimuli in patients was not due to an inability to perceive HSF. The LSF preference found in schizophrenia confirms our previous study conducted with faces, and shows that this LSF bias generalizes to other categories of objects. When a broad range of spatial frequencies are present in the image, as in normal conditions of viewing, patients preferentially rely on coarse visual information contained in LSF. This result may be interpreted as a dysfunction of the guidance of HSF processing by LSF processing.

## Introduction

How do patients with schizophrenia use visual information to form a coherent representation of the world? Schizophrenia does not only impair high-level cognitive functions, such as executive functions (Dickinson et al., 2007), emotion recognition (Morris et al., 2009), or theory of mind (Sprong et al., 2007). It also impairs the processing of low level perceptual information such as spatial frequencies (Slaghuis, 1998; Butler et al., 2005). Visual spatial frequencies are often considered as the atomic element of perception (Valois and Valois, 1990). The lowest spatial frequencies (below 1.5 cycle/degree) contain a coarse representation of the visual stimuli and are preferentially conveyed by the magnocellular pathway. The detailed information contained in high spatial frequencies (HSF) is primarily processed by the parvocellular pathway (Kaplan and Shapley, 1986).

Several studies suggest that this early processing may be dysfunctional in schizophrenia: for instance, patients have poorer performance in contrast sensitivity tasks at different spatial frequency ranges, compared to healthy controls (Slaghuis, 1998, 2004; Kéri et al., 2002; Butler et al., 2005, 2007; Revheim et al., 2006; Kéri and Benedek, 2007; Martínez et al., 2008; Kantrowitz et al., 2009). Other studies have found a specific deficit of the processing of very low spatial frequencies (LSF), below 1.5 cycle/degree (Butler and Javitt, 2005; Revheim et al., 2006; Butler et al., 2007), which has been linked to subcortical magnocellular dysfunction (Butler and Javitt, 2005), although this interpretation remains controversial (Skottun and Skoyles, 2007).

Beside their implication in early visual processing, spatial frequencies also play a role in the formation of coherent visual representations. In realistic conditions of viewing, our visual system is exposed to a very large range of spatial frequencies and preferentially selects some spatial scales over others depending on temporal processing (e.g., exposure duration, Schyns and Oliva, 1994; Oliva and Schyns, 1997; Peyrin et al., 2006), task requirements (Schyns and Oliva, 1999; Peyrin et al., 2005), or distance of viewing (Brady and Oliva, 2012). In a previous study, our team has explored how different spatial frequencies are integrated to form a coherent representation of a face in patients with schizophrenia (Laprévote et al., 2010). We measured performance in a facial expression recognition task with hybrid faces. Hybrid faces are images made of two superimposed faces, one face filtered to only keep the LSF and the other filtered to only keep HSF. The results showed that patients suffering from schizophrenia more often used the LSF to recognize facial expression at a glance. A control experiment with single faces shown either in LSF or in HSF demonstrated that patients were able to process both spatial scale components when shown separately, meaning they could see very well the details of the HSF percept, when not in competition with a LSF percept. Therefore, the bias for LSF in hybrid images was interpreted as a deficit of the mechanism of spatial frequency integration in the brain.

One caveat of our previous study however, is that we used a facial expression recognition task, which might have introduced additional biases leading to the appearance of a HSF impairment. Faces are considered as special visual objects which activate specific brain networks (Kanwisher and Yovel, 2006; Atkinson and Adolphs, 2011). This specificity implies a differential processing of spatial frequencies for face stimuli: the optimal spatial band for face recognition is comprised between 8 and 16 cycles/face, so on the lower end of the spatial spectrum (Costen et al., 1996), and LSF may play a more significant role than HSF in rapid face processing because LSF support configural processing (Goffaux et al., 2005) and precede the integration of HSF (Awasthi et al., 2011). Moreover, it is known that people with schizophrenia have an emotion recognition deficit (Morris et al., 2009), and that the emotional content of a face modulates spatial frequency perception (Smith and Schyns, 2009; Kumar and Srinivasan, 2011). In a study using bubbles technique during a facial emotion discrimination task, Lee et al. (2011) have shown that patients with schizophrenia had an atypical strategy of using visual information to recognize different emotions: patients relied less frequently less frequently on high frequency information contained in the eyes of the fearful faces, whereas they used higher spatial frequencies to recognize happiness.

**Figure 1:**
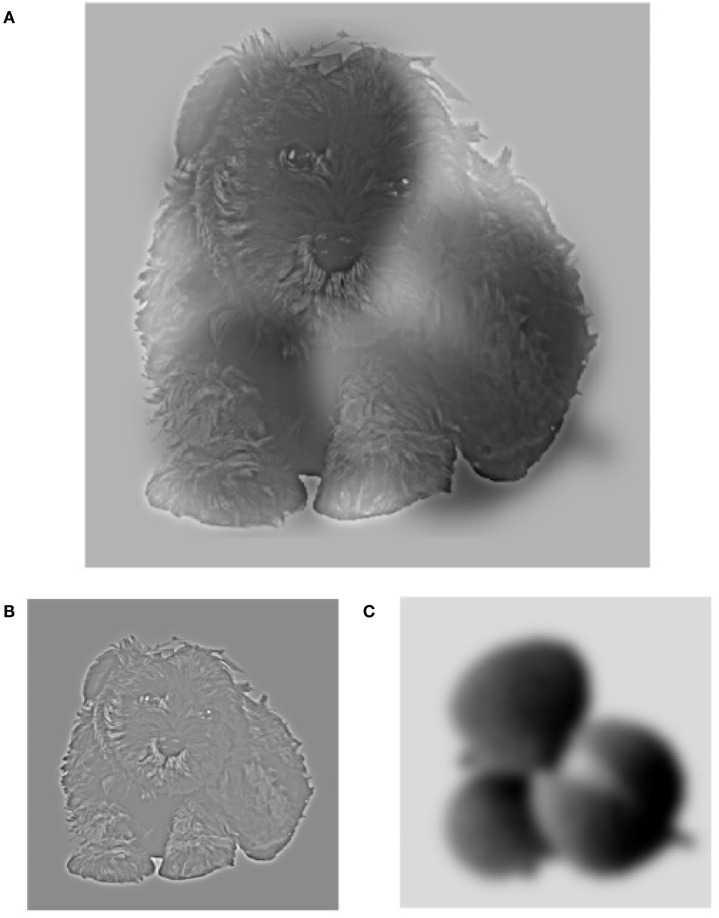
**Example of a hybrid stimulus (A)**. The hybrid combines the high spatial frequency information from the baby dog in image **(B)** with the low spatial frequency information from the litchi in image **(C)**. The high spatial frequency component of the hybrid can be seen more easily if you hold the image close to your eyes, and the low spatial frequency information can be better seen if you step away from the image or slightly blur your gaze. Image **(B,C)** are examples of filtered images that were used in the control experiment.

To further investigate the processing of rapid spatial frequency integration in patients with schizophrenia, we ran two psychophysical experiments with objects instead of faces. Is the bias found for low spatial frequency in hybrid stimuli (Laprévote et al., 2010) face specific or a generic mechanism of object recognition? In the first experiment we showed hybrid stimuli (Figure 1A) for a short glance, each made of a picture of an animal and a picture of a fruit, one in LSF and the other in HSF. With this simple design, the image category reported by the participant directly tells the spatial scale preferentially perceived. Accuracy results showed that patients with schizophrenia reported significantly more frequently the LSF image than the control group of participants. A control experiment tested the origin of the LSF bias in Experiment 1: all participants performed the same object categorization task but were shown filtered single pictures of animals or fruits, showing only the HSF or the LSF components of each image (Figure 1B). This allowed us to determine whether the LSF bias observed in the first experiment was due to a specific deficit at processing HSF or a deficit in concurrently processing HSF and LSF in images containing a broad spectrum of spatial frequencies (as in normal perception).

## Main Experiment

### Method

#### Participants

Fifteen adult individuals suffering from schizophrenia were recruited from the Public Mental Health Institute of Lille Metropole (Armentières, France), the Public Mental Health Institute of Flandres (Bailleul, France), and the Department of General Psychiatry in Lille University Hospital (Lille, France). The inclusion criteria required an age of 18–55 years and a diagnosis of schizophrenia based on standard DSM-IV criteria (American Psychiatric Association, 2000). All participants’ visual acuity was measured by the Snellen chart. Only patients and controls with a normal or corrected-to-normal visual acuity (10/10 on Snellen chart) were included. The exclusion criteria were history of neurological illness, trauma occurring in the past 6 months, ophthalmic illness, and alcohol or drug abuse. All patients received antipsychotic medication and were clinically stable at testing time. Symptoms of schizophrenia were assessed with the Positive and Negative Syndrome Scale (PANSS) (Kay et al., 1987). Twelve age and gender-matched healthy controls were initially recruited. They were free from a DSM-IV axis-I diagnosis and reported taking no medication. The study was approved by the Ethics Committee of Lille University Hospital. A written consent was obtained from all participants. No participant was paid for taking part in the study.

#### Stimuli

Hybrid stimuli were made of an animal and a fruit. The original picture set was taken from the Hemera Photo Object database. In that set, 35 animals and 35 fruits were grouped by pairs and were aligned so that inner and outer object characteristics overlapped (see Figure 1). The pictures grayscale images, centered in a matrix of 256 × 256 pixels. We created a low-pass version (below 8 cycles/image) and a high-pass version (above 24 cycles/image) of each object (see examples in Figure 1), for a total of 70 HSF-only objects and 70 LSF-only objects. Then, we created hybrid stimuli by overlapping a low-pass filtered object of one of the categories (animal/fruit) with the corresponding high-pass filtered object of the other category. Each pair of objects provided two hybrids images (LSF fruit/HSF animal and HSF fruit/LSF animal). As each hybrid was composed of two different objects, an animal and a fruit, the participant’s response on a given hybrid image indicated which spatial scale was preferentially reported.

#### Procedure

Participants were seated in a darkened room with their head stabilized by a chin-rest at a viewing distance of 140 cm. Stimuli subtended 2.5° of visual angle. A central fixation cross was shown for 1 s, followed by a hybrid stimulus displayed for 100 ms. This presentation time was chosen to allow only one fixation on the stimulus, as the average human gaze fixation is around 300 ms (Harris et al., 1988). The stimuli were presented in a randomized order. Participants were asked to decide verbally whether the object was an animal or a fruit. The answer was coded by the experimenter on the keyboard of the computer. Before this experiment, participant performed a practice trial with five hybrids stimuli, in order to familiarize with the task. This hybrid set was different from the experimental set. On the basis of a pilot study on healthy participants, eight hybrid images were excluded in the final analysis because one of the two images proved to be more salient in terms of luminance.

#### Statistical analysis

Statistical analyses were conducted with STATISTICA 6.1 software (StatSoft Inc.).

The main dependent variable was the percentage of object categorization responses to images for each spatial frequency component. Upon examination of the data distribution, we found that one healthy control’s data were two standard deviations above the mean, so we did not include that data in the statistical analysis. The percentage of responses in each spatial frequency was compared between patients and healthy controls with a bilateral *t*-test.

Two-tailed Pearson correlations were used to check any relationship between the percentage of responses in each spatial frequency and antipsychotic daily dose, benzodiazepine dose, age, or PANSS dimensions.

### Results

The characteristics of the population are summarized in Table 1. Ages of patients and healthy controls were respectively 36.8 (SEM = 2.8) and 35.3 (SEM = 2.2) years.

**Table 1:** **Population characteristics**.

	Healthy controls (*N* = 11) Mean (SEM)	Patients with schizophrenia (*N* = 15) Mean (SEM)
Age (years)	36.8 (2.8)	35.3 (2.2)
Gender (male/female)	8 M/3 F	12 M/3 F
Antipsychotic medication (mg chlorpromazine Eq)		688 (162)
Benzodiazepine medication (mg diazepam Eq)		29 (10)
PANSS total score		75.7 (3.5)
PANSS positive symptoms		13.4 (1)
PANSS negative symptoms		26.4 (1)
PANSS general psychopathology		35.8 (2)

Our main measure was the percentage of responses in each spatial frequency.

For hybrid stimuli, the percentage of responses based on the LSF object was respectively 50.67% (standard error of the mean, SEM, was 5.89%) for patients and 32.87% (SEM 5.55%) for healthy controls. This difference was significant [*t*(24) = 2.12, *p* < 0.05]. The complementary percentage of responses based on HSF was 49.32% (SEM 5.89%) for patients and 67.12% (SEM 5.55%) for healthy controls. As there was a small number of participants, a *post hoc* power analysis was conducted with the software G_Power3 (Faul et al., 2007). It showed that the power of our design was 0.68 (α = 05).

We did not find any significant correlation between the percentage of responses, in each spatial frequency and antipsychotic daily dose, benzodiazepine dose, age, or any PANSS dimension.

## Control Experiment: High and Low Spatial Frequency Filtered Objects

### Aim

This control experiment verifies if participants could perform the main task correctly and assesses if the bias found with hybrid images in main experiment resulted from an inability to process HSF components. To do so, filtered images containing only LSF or only HSF were used.

### Method

#### Participants

The same two populations performed the control experiment. This experiment was conducted after Experiment 1, after a 10 min pause, in the same conditions.

#### Stimuli

We used the same original animals and fruits sets as in those used with hybrid stimuli. We created a low-pass version (below 8 cycles/image) and a high-pass version (above 24 cycles/image) of each object (see examples in Figure 1), for a total of 70 HSF-only object images (35 animals and 35 fruits) and 70 LSF-only object images (35 animals and 35 fruits).

#### Procedure

This set of 140 images was presented to participants in the same conditions as in experiment 1 (distance was 140 cm, size of 2.5° of visual angle, and stimulus presentation time was 100 ms). Participants responded orally if the picture was an animal or a fruit, and the answer was coded by the experimenter on the keyboard of the computer. The 140 stimuli were presented in a randomized order in two blocks of 70 trials, separated by a short pause.

#### Statistical analysis

We collected the percentage of correct responses in each spatial frequency. This variable was analyzed with a repeated measures ANOVA with “group” as between factor and “spatial frequency” and “object category” as within factors.

### Results

Healthy controls correctly reported the category in 96.6% (SEM 0.6%) for HSF-only images and 94.7% (SEM 0.8%) for LSF-only images. Patients’ responses were 91.9% (SEM 1.9%) for HSF-only images and 91.0% (SEM 1.5%) for LSF-only images. The ANOVA showed a main effect of group [*F*(1, 24) = 4.65, *p* < 0.05), as on average healthy controls were more accurate than patients. The ANOVA revealed no effect of spatial frequency or stimulus category on correct answers. There was no spatial frequency × group interaction. Spatial frequency interacted with category [*F*(1, 24) = 7.26, *p* = 0.01]. This interaction resulted from fruits being better recognized in high spatial frequency than in low spatial frequency [*F*(1, 24) = 5.86, *p* < 0.05] whereas there was no significant difference for animals. There was no spatial frequency × category × group interaction.

## Discussion

The goal of this study was to verify if the low spatial frequency bias previously observed in schizophrenia (Laprévote et al., 2010) is face specific or generalizes to other object categories. Patients suffering from schizophrenia and healthy controls performed an object categorization task on hybrid stimuli, which combined an animal and a fruit, shown each at a different spatial frequency range. In the hybrids, patients categorized more frequently the images in low spatial frequency than did healthy controls. We verified that the LSF bias was not due to a task misunderstanding or to a deficit in the processing of HSF components as both patients and controls categorized objects with high accuracy when HSF-only and LSF-only stimuli were used.

This result replicates previous findings with hybrid faces, demonstrating a strong bias toward low spatial frequency in schizophrenia when categorizing images at a glance (Laprévote et al., 2010). This LSF bias contrasts with the predictions made on the basis of the subcortical magnocellular deficit hypothesis in schizophrenia (Butler and Javitt, 2005). Because LSF are preferentially conveyed by the magnocellular pathway, such a deficit would imply a deficit of the perception of LSF. However, our results challenge this view, and suggest a framework where spatial frequencies are integrated in a dynamic fashion to form an object percept. Associating high and LSF is crucial for the recognition of complex visual stimuli: LSF are processed early on by the visual system and they precede the slower integration of HSF to form a full spatial scale percept (Schyns and Oliva, 1994; Bar, 2004). In our two studies, patients with schizophrenia based their decision on default LSF information, neglecting the potentially slower processing of HSF. Indeed, Bar et al. (2006) (Kveraga et al., 2007) have proposed that coarse visual information contained in LSF are quickly carried over by the dorsal cortical pathway and can rapidly provide a skeleton layout of visual information to orbito-frontal cortex, which influences by feedback connections the slower processing of details conveyed by HSF preferentially processed in the ventral cortical pathway. Our current results fit well with a dysfunction of spatial scale integration, in line with other studies suggesting a dysfunction of the interaction between dorsal and ventral pathways in patients with schizophrenia (Doniger et al., 2002; Schechter et al., 2003; Foxe et al., 2005; Ducato et al., 2008; Plomp et al., 2012). In addition, recent findings have also suggested that a dysfunction of early processing by the dorsal stream may impair high-level visual functions. For instance, Sehatpour et al. (2010) examined cortical activations with fMRI during the formation of a coherent object representation via a perceptual disclosure paradigm in patients with schizophrenia. Patients had less activity overall of the dorsal visual network which contributed to subsequent impaired activity of the ventral visual stream. Our results are also aligned with this proposal: coarse information contained in LSF may be processed at a minimum, but the signal strength may not be sufficient to allow amplification of subsequent detailed processing, implying a preference for LSF visual information.

A possible limitation to our study is that patients were under medication at the time of testing. Benzodiazepines have been shown to impair contrast sensitivity, mainly for LSF (Haris and Phillipson, 1995). Also, antipsychotics have been shown to impair contrast perception for HSF and to increase contrast perception for LSF (Harris et al., 1990). While our analysis failed to find any significant correlation between spatial frequency preference and benzodiazepine or antipsychotics daily dose, further studies will be necessary to measure the impact of those treatments on spatial frequency preference. The absence of any experiment testing directly performances of participant with hybrid faces may also constitute a bias. This methodological choice has been made in order to simplify experimental design and preserve attentional capacities of participants. However, we conducted the experiments of this paper in the same experimental condition as our previous work about hybrid faces (Laprévote et al., 2010). At last the small sample sizes may also constitute a limitation of this study.

To summarize, here we confirm that patients suffering from schizophrenia have a preference for coarse LSF information in fast visual categorization tasks, and show that this preference is not specific to faces but generalizes to other categories of objects. As patients, like controls, performed well with separated LSF and HSF stimuli, the LSF bias when processing full range hybrid images, as in normal conditions of viewing, may be interpreted as a dysfunction of the integration of spatial scales, a fundamental mechanism to form a rich and coherent representation of visual objects. Schizophrenia patients may preferentially rely on default LSF information to appreciate their visual environment.

## Conflict of Interest Statement

The authors declare that the research was conducted in the absence of any commercial or financial relationships that could be construed as a potential conflict of Interest.
